# Factors influencing participant enrolment in a diabetes prevention program in general practice: lessons from the Sydney diabetes prevention program

**DOI:** 10.1186/1471-2458-12-822

**Published:** 2012-09-24

**Authors:** Rachel A Laws, Philip Vita, Kamalesh Venugopal, Chris Rissel, Daniel Davies, Stephen Colagiuri

**Affiliations:** 1 Prevention Research Collaboration, School of Public Health, University of Sydney, New South Wales, 2006, Australia; 2 Boden Institute of Obesity, Nutrition, Exercise & Eating Disorders, Sydney Medical School, University of Sydney, New South Wales, 2006, Australia

**Keywords:** Diabetes prevention, Program participation, Enrolment, Risk factors, Research translation

## Abstract

**Background:**

The effectiveness of lifestyle interventions in reducing diabetes incidence has been well established. Little is known, however, about factors influencing the reach of diabetes prevention programs. This study examines the predictors of enrolment in the Sydney Diabetes Prevention Program (SDPP), a community-based diabetes prevention program conducted in general practice, New South Wales, Australia from 2008–2011.

**Methods:**

SDPP was an effectiveness trial. Participating general practitioners (GPs) from three Divisions of General Practice invited individuals aged 50–65 years without known diabetes to complete the Australian Type 2 Diabetes Risk Assessment tool. Individuals at high risk of diabetes were invited to participate in a lifestyle modification program. A multivariate model using generalized estimating equations to control for clustering of enrolment outcomes by GPs was used to examine independent predictors of enrolment in the program. Predictors included age, gender, indigenous status, region of birth, socio-economic status, family history of diabetes, history of high glucose, use of anti-hypertensive medication, smoking status, fruit and vegetable intake, physical activity level and waist measurement.

**Results:**

Of the 1821 eligible people identified as high risk, one third chose not to enrol in the lifestyle program. In multivariant analysis, physically inactive individuals (OR: 1.48, P = 0.004) and those with a family history of diabetes (OR: 1.67, P = 0.000) and history of high blood glucose levels (OR: 1.48, P = 0.001) were significantly more likely to enrol in the program. However, high risk individuals who smoked (OR: 0.52, P = 0.000), were born in a country with high diabetes risk (OR: 0.52, P = 0.000), were taking blood pressure lowering medications (OR: 0.80, P = 0.040) and consumed little fruit and vegetables (OR: 0.76, P = 0.047) were significantly less likely to take up the program.

**Conclusions:**

Targeted strategies are likely to be needed to engage groups such as smokers and high risk ethnic groups. Further research is required to better understand factors influencing enrolment in diabetes prevention programs in the primary health care setting, both at the GP and individual level.

## Background

Diabetes has been recognised as a significant contributor to global disease burden [1]. The rising prevalence of diabetes worldwide, coupled with the evidence of the costs and complications associated with this condition means prevention is an important strategy to reduce disease burden [1,2].Obesity, physical inactivity and poor nutrition are major modifiable lifestyle risk factors for diabetes, making lifestyle interventions an obvious area to target for diabetes prevention [1].

The efficacy of intensive lifestyle interventions in preventing or delaying the onset of diabetes amongst high risk individuals has been well established in a number of large randomised controlled trials [3-7]. Meta-analyses of these trials has shown that lifestyle intervention can reduce the incidence of diabetes by around 50% [8] and is at least as effective as drug treatment [9]. There are, however, a number of challenges in translating the findings of these large trials to achieve population health benefits. The first challenge is how to deliver such programs in a sustainable way in the community context and as part of routine service delivery. The second challenge is how best to engage high risk individuals to participate in such interventions.

Recent replication trials have demonstrated that it is feasible to implement community based diabetes prevention programs and early results have been promising [10-17]. However, the reach of diabetes prevention programs has rarely been reported in the literature. A number of efficacy and replication trials have not provided any information on enrolment rates amongst eligible participants [4,6,12,18-23]. In other trials the proportion of eligible participants who agreed to enroll has varied widely from a third to 100 percent [3,5,7,10,11,13-16]. Even less is known about factors influencing enrolment in such programs. Understanding factors influencing enrolment is critical to improving the reach and population health impact of diabetes prevention programs.

This paper examines the predictors of enrolment in a diabetes prevention program conducted in general practice in New South Wales (NSW), Australia from 2008 to 2011. The findings will provide new insights into those individuals who are more or less likely to enrol in diabetes prevention programs informing the development of targeted recruitment strategies, particularly in the primary health care setting.

## Methods

### Study context

This research is part of a larger study, the Sydney Diabetes Prevention Program (SDPP), the details of which have been published elsewhere [24]. In brief, SDPP is a community-based translation study of diabetes prevention which aims to assess the effectiveness of a lifestyle modification program on modifiable risk factors for type 2 diabetes. The study is being conducted in three Divisions of General Practice (two urban and one rural area) in NSW, Australia. The Divisions of General Practice have recruited over 75 practices and 150 general practitioners (GPs) to participate in the study. This was done through expression of interest by letter and fax, information sessions and site visits. The main pre-requisite for inclusion was the practice having a computerised patient record system.

### Participant recruitment

Participating GPs approached individuals aged 50–65 years without known diabetes to participate in the program. Potential participants were identified by practice staff using a variety of methods, including opportunistic recruitment during routine consultations, sending letters of invitation to patients in the target age range and through local media promotion. Potential participants were screened using the Australian Type 2 Diabetes Risk Assessment (AUSDRISK) Tool (Table 1), a validated predictor of diabetes risk at five year follow up [25]. Individuals at high risk of diabetes (AUSDRISK score ≥ 15) were invited to participate after diabetes has been excluded. Other exclusion criteria included taking hypoglycaemic medication in the past month, use of prescribed weight loss medication or medical contraindication to participate in physical activity. The lifestyle modification program consisted of an initial individual health coaching session and three group sessions (or individual telephone coaching sessions) over the first three months, followed by three monthly follow up telephone health coaching contacts over 12 months. The program was based on behaviour change principles and focuses on five goals: 5% weight loss, 210 min/week physical activity, limit total dietary fat and saturated fat to less than 30% and 10% of energy respectively and at least 15 g/1000 kcal dietary fibre. Primary outcomes of changes in weight, physical activity and diet were assessed at 12 months along with secondary outcomes including changes in waist circumference, fasting plasma glucose, lipids, quality of life, psychological well being, medication use and health service utilisation.

**Table 1:** The Australian Type 2 Diabetes Risk Assessment Tool

		**Your Score**				**Your Score**
**1. Your age group?**			**6. Are you currently taking medication for high blood pressure?**			
Under 35 years	0 points		No		0 points	
35 – 44 years	2 points		Yes		2 points	___
45 – 54 years	4 points					
55 – 64 years	6 points					
65 years or over	8 points	___				
**2. Your gender?**			**7. Do you currently smoke cigarettes or any other tobacco products on a daily basis?**			
Female	0 points		No		0 points	
Male	3 points	___	Yes		2 points	___
**3. Ethnicity/Country of birth:**			**8. How often do you eat vegetables or fruit?**			
3a. Are you of Aboriginal, Torres Strait Islander, Pacific Islander or Maori descent?			Everyday		0 points	
No	0 points		Not everyday		1 point	___
Yes	2 points	___	**9. On average, would you say you do at least 2.5 hours of physical activity per week (for example, 30 minutes a day on 5 or more days a week)?**			
3b. Where were you born?			Yes		0 points	
Asia (including the Indian sub-continent), Middle East, North Africa, Southern Europe	2 points		No		2 points	___
Other	0 points	___				
**4. Have either of your parents, or any of your brothers or sisters been diagnosed with diabetes (type 1 or type 2)?**			**10. Your waist measurement taken below the ribs (usually at the level of the navel)?**			
No	0 points		**For those of Asian or Aboriginal or Torres Strait Islander descent:**			
Yes	3 points	___	Men	Women		
**5. Have you ever been found to have high blood glucose (sugar) (for example, in a health examination, during an illness, during pregnancy)?**			Less than 90 cm	Less than 80 cm	0 points	
No	0 points		90 – 100 cm	80 – 90 cm	4 points	
Yes	6 points	___	More than 100 cm	More than 90 cm	7 points	
			**For all others:**			
			Men	Women		
			Less than 102 cm	Less than 88 cm	0 points	
			102 – 110 cm	88 – 100 cm	4 points	
			More than 110 cm	More than 100 cm	7 points	___
	**Subtotal**		**Subtotal**			
Your risk of developing Type II diabetes within 5 years *:			**Total Risk Score**			
6 - 11: Increased risk			Increased risk of developing type 2 diabetes			
12 or more: High risk			May have undiagnosised type 2 diabetes or be at high risk of developing type 2 diabetes			

* The overall score may overestimate the risk of diabetes in those aged less than 25 years and underestimate the risk of diabetes in people of Aboriginal and Torres Strait Islander descent.

### Data collection

The AUSDRISK tool [25] was completed by all potential participants who consented. The 10 item tool comprised demographic questions including age, gender, ethnicity, country of birth and other known risk factors for diabetes such as family history of diabetes, previous history of high blood glucose, smoking status, fruit and vegetable intake, physical activity levels as well as an objective assessment of waist circumference performed by the GP or practice staff. Following the screening process, the eligibility status of participants was recorded by practice staff and eligible participants invited to attend the program.

 The postcode of residence for each potential participant was also recorded and linked to the 2006 index of relative socio-economic advantage/disadvantage [26] for the area in which the patient lived. The index ranks geographical areas where a high proportion of people are relatively more, or less, disadvantaged taking intoaccount income, education, occupation, wealth and living conditions. A lower score indicates that an area is relatively disadvantaged compared with an area with a higher score. The index was linked to the patients’ postcode of residence using quintiles. A quintile number of one represented the lowest 20% of areas, up to the highest 20% of areas which were given a quintile number of five. For the purposes of analysis three categories were created: 1) most disadvantaged patients (quintiles one and two), 2) intermediate disadvantaged patients (quintile three) and 3) least disadvantaged patients (quintiles four and five).

### Data analysis

The dependent (response variable) of interest was enrolment outcomes. The enrolment outcomes for potential participants were recorded (ineligible, eligible and enrolled, eligible and did not enrol). Enrolment was defined as having attended the initial intervention session – the individual consultation. Univariate analysis was initially undertaken to compare the characteristics of eligible participants who enrolled in the program and those who did not (ineligible participants were excluded from the analysis). This consists of chi-square analysis for categorical variables, independent sample T test for normally distributed continuous variables and Mann–Whitney U test for non parametric continuous variables. There was a significant association between enrolment outcomes by GP (chi square = 372.65, P = 0.000), however the Intra Class Correlation Coefficient was not significant (ICC = 0.003, P = 0.477). All variables identified as significant in the univariate analysis were entered into a multivariate model using generalized estimating equations (computed using the full log quasi-likelihood function and logit link function) to control for the small clustering effect. All analysis was undertaken using SPSS version 17.0.

### Ethics

Ethics approval to conduct this trial was granted by the Research Ethics Review Committee of Sydney South West Area Health Service (ID Number X08-0053). Written informed consent was obtained from all participants.

## Results

**Figure 1:**
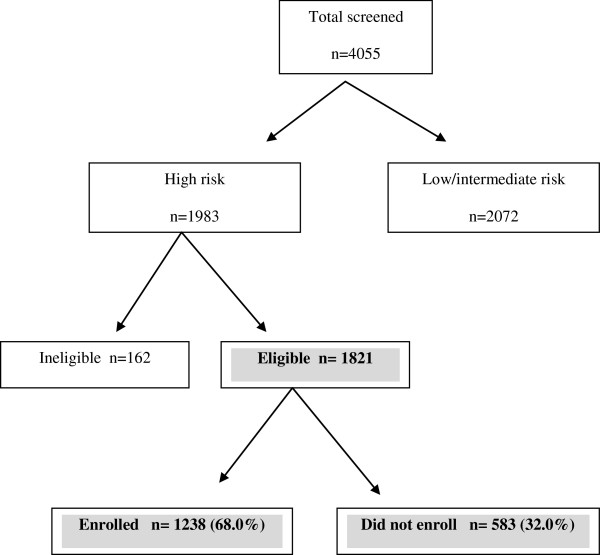
Participant recruitment and enrolment.

A total of 4055 individuals were screened, of which 1821 were eligible to participate in the study. Approximately two-thirds of eligible participants (n = 1238) enrolled in the program (Figure 1). The number of individuals enrolled by each GP varied considerably, with over a third of GPs (n = 77) not recruiting any and the majority (58.8%) enrolling between 1–20 participants. The mean AUSDRISK score of enrolled individuals was significantly higher than those who chose not to enrol in the program. Enrolled participants were more likely to have a family history of diabetes or history of high glucose levels and be physically inactive. However, individuals who did not enrol in the program were more likely to be male, of Aboriginal or Torres Strait Islander background, born in a country with high diabetes risk, smoke, take blood pressure lowering medication and consume low amounts of fruit and vegetables (Table 2). Interestingly, waist circumference, age and socioeconomic status (SEIFA index) were not associated with enrolment rates.

**Table 2:** Characteristics of eligible participants who enrolled compared with eligible participants who did not enrol in the program

	**Did not enroll n= 583 No (%)**	**Enrolled n=1238 No (%)**	**Significance**
**Socio-demographic Characteristics**			
**Gender**			
Female	319 (54.7)	774 (62.6)	**P=0.001**
Male	264 (45.3)	462 (37.4)	
**Age**			
45-54 years	138 (23.7)	288 (23.3)	P=0.672
55-64 years	414 (71.0)	871 (70.4)	
65 years and over	31 (5.3)	79 (6.4)	
**Aboriginal, Torres Strait Islander, Pacific Islander or Maori descent**			
Yes	37 (6.5)	44 (3.6)	**P=0.008**
No	536 (93.5)	1157 (96.3)	
**Region of Birth**			
Asia (including Indian sub-continent), middle east, North Africa, Southern Europe	117 (20.6)	164 (13.4)	**P=0.000**
Australia or other	451 (79.4)	1063 (86.6)	
**Socioeconomic status (SEIFA index)**			
Most disadvantaged (SEIFA quintile 1-2)	21 (3.7)	40 (3.2)	P=0.629
Intermediate Deprivation (SEIFA quintile 3)	103 (18.1)	202 (16.4)	
Least disadvantaged (SEIFA quintile 4- 5)	449 (78.4)	987 (80.3)	
**Participant Health Risk Profile Family history of type 1 or type 2 diabetes**			
Yes	208 (36.4)	584 (47.7)	**P=0.000**
No	363 (63.6)	641 (52.3)	
**History of high blood glucose**			
Yes	203 (35.5)	552 (45.2)	**P=0.000**
No	369 (64.5)	668 (54.8)	
**Taking antihypertensive medication**			
Yes	326 (56.7)	622 (50.4)	**P=0.013**
No	249 (43.3)	611 (49.6)	
**Daily Smoker**			
Yes	108 (18.8)	137 (11.1)	**P=0.000**
No	467 (81.2)	1094 (88.9)	
**Frequency of fruit and vegetable consumption**			
Every day	431 (75.1)	990 (80.4)	**P=0.011**
Not every day	143 (24.9)	242 (19.6)	
**Physical activity levels**			
At least 2.5 hours per week	314 (54.5)	573 (46.6)	**P=0.002**
Less than 2.5 hours per week	262 (45.5)	657 (53.4)	
**Waist measure, mean (SD), n**	105.8 (13.2), 469	104.5 (12.1) 1015	P=0.101
**Total risk score, mean (SD), median**	18.4 (3.0), 18	18.8 (3.4), 18	**P=0.029**

All of the factors found to be significant predictors of enrolment in univariate analysis remained independent predictors of enrolment in the multivariate model after adjusting for other variables and clustering by GPs, with the exception of gender (OR: 0.80, P = 0.100) and Aboriginal and Torres Strait Islander decent (OR: 0.53, P = 0.068), which were no longer significant predictors of enrolment (Table 3). In particular, physically inactive individuals (OR: 1.48, P = 0.004), those with a family history of diabetes (OR: 1.67, P = 0.000) and those with a history of high blood glucose levels (OR: 1.48, P = 0.001) were significantly more likely to enrol in the program. However, high risk individuals who smoked (OR: 0.52, P = 0.000), were born in a country with high diabetes risk (OR: 0.52, P = 0.000), were taking blood pressure lowering medications (OR: 0.80, P = 0.040) and consumed little fruit and vegetables (OR: 0.76, P = 0.047) were significantly less likely to take up the program. Overall, the model was able to correctly classify 69.7% of individuals, suggesting that the variables in the model are useful in accounting for the variation in enrolment rates.

**Table 3:** Generalized Estimating Equations (GEE) model results for enrolment in the program (adjusted for clustering by GP)

**Variable**	**OR (CI)**	**P Value (adjusted for clustering by GP)**
**Gender**		
Female	1.00(ref)	P=0.100
Male	0.802 (0.616-1.044)	
**Aboriginal, Torres Strait Islander, Pacific Islander or Maori descent**		
No	1.00(ref)	
Yes	0.526 (0.263-1.049)	P=0.068
**Region of Birth**		
Australia or other	1.00 (ref)	**P=0.000**
Asia (including Indian sub-continent), middle east, North Africa, Southern	0.520 (0.375-0.721)	
Europe		
**Participant Health Risk Profile Family history of type 1 or type 2 diabetes**		
No	1.00 (ref)	**P=0.000**
Yes	1.665 (1.351-2.051)	
**History of high blood glucose**		
No	1.00 (ref)	**P=0.001**
Yes	1.484 (1.175-1.874)	
**Taking antihypertensive medication**		
No	1.00(ref)	**P=0.040**
Yes	0.798(0.644-0.990)	
**Daily Smoker**		
No	1.00(ref)	**P=0.000**
Yes	0.517 (0.400-0.669)	
**Frequency of fruit and vegetable consumption**		
Every day	1.00(ref)	**P=0.047**
Not every day	0.760 (0.579-0.996)	
**Physical activity levels**		
At least 2.5 hours per week	1.00(ref)	**P=0.004**
Less than 2.5 hours per week	1.479 (1.134-1.930)	

## Discussion

This study provides important new insights into the factors influencing enrolment in diabetes prevention programs. Approximately one third of eligible individuals chose not to enrol in the lifestyle modification program, despite undergoing the initial screening process. Our findings suggest that some high risk individuals including those with a family history of diabetes, previous history of high blood glucose and physically inactive individuals are more likely to enrol in diabetes prevention programs. However, other high risk individuals including smokers, those born in a country with high diabetes risk, individuals taking blood pressure lowering medications and low consumers of fruit and vegetables are significantly less likely to take up such programs.

To our knowledge, few previous diabetes prevention studies have examined predictors of enrolment. In line with our findings, The DE-PLAN study in Greece [16] reported that program participation was independently associated with glucose intolerance and the site of recruitment. In contrast to our findings, Narayan et al. [27] found that after controlling for age, men were more likely to decline to take part compared with women, and after controlling for age and sex, people declining were more likely to have a lower weight and waist circumference. Little information was provided about other variables included in the models, making direct comparisons difficult.

The region of birth of participants was the only measured socio-demographic characteristic predictive of enrolment rates in our study. Despite their higher diabetes risk, individuals born in the Indian sub-continent, Middle East, Southern Europe and North Africa were less likely to enrol in the program. This highlights the importance of having programs targeting high risk ethnic groups as these groups are less likely to enrol in mainstream programs. In line with this, intervention programs specifically targeting Arabic-speaking and Chinese-speaking people were provided as part of the SDPP [24].

While indigenous status and socio-economic status (SES) were not independent predictors of enrolment in this study, this may reflect a lack of power to detect an effect due to the small number of eligible participants of Aboriginal and Torres Strait Islander descent in the study (n = 81, 4.4%) and the limited variation in the SES of participants, with few participants (n = 61, 3.4%) from areas of high deprivation. Given the high prevalence of diabetes amongst Aboriginal and Torres Strait Islanders [28] and the poorer health status of low SES groups [29], further research is required to explore uptake of diabetes prevention programs in these populations. Finally, the lack of association between age and enrolment likely reflects the narrow age range for this study (50–65 years) and the limited age categories used in the AUSDRISK tool.

Our findings highlight the importance of an individual’s health risk profile in predicting enrolment rates in diabetes prevention programs. A family history of diabetes and a previous history of high blood glucose were strong independent predictors of program uptake, suggesting high awareness and motivation amongst these individuals. Similarly, those individuals not meeting physical activity recommendations of 2.5 hours of physical activity per week were also more likely to enrol in the program. .While the measure of physical activity was crude, consisting of a single question (*“On average, would you say you do at least 2.5 hours of physical activity per week (for example, 30 minutes a day on 5 or more days a week)?)* this has been found to be a significant predictor of diabetes risk. Given that family history, of high blood glucose and physical inactivity are important risk factors for diabetes [25] our findings highlight the potential value in targeting those high risk individuals who are likely to be most receptive. In contrast, smokers were significantly less likely to take up the program, despite their higher diabetes risk [30]. There is some evidence to suggest that smokers are less likely to participate in health promotion programs [31,32], and less likely to participate in research studies [33,34].

Low consumers of fruit and vegetables (less than daily) were also less likely to take up the program. It should be noted that the measure of fruit and vegetable consumption was crude (‘everyday’ versus ‘not every day’) and may not accurately reflect actual intake. Finally individuals taking anti-hypertensive medication were also less likely to enrol. The reason for this is uncertain. As few studies have examined diabetes risk perception and its relationship with enrolment in diabetes prevention programs, these findings require exploration in future research.

While primary health care has been identified as an important setting for chronic disease prevention [35], the overall low rates of enrolment by GPs in this study points to the difficulties in implementing these programs in general practice, raising the question of how best to engage GPs in prevention. It could also be that since the AUSDRISK tool had only recently been developed and implemented as a screening tool, there was limited awareness and use in general practice [36]. The low rates of engagement by GPs may reflect the fact that the program was a trial and not an ongoing service. Research suggests that better system support is required to engage general practice in prevention including adequate funding and reimbursement systems, the use of staff such as practice nurses and managers and referral brokers (who act to facilitate referrals between GPs and services), along with better practice systems such as patient registration and recall and reminder systems [37-41]. Our findings also highlight the importance of appropriately briefing GPs on prevention programs so they can encourage participation amongst their high risk patients. The provision of prompt feedback to GPs on the progress of referred individuals is also likely to be important in encouraging future referrals.

This study has a number of limitations. Firstly, data were not collected on the total number of participants approached and the proportion who agreed to be screened. It is likely that a high proportion of individuals who were approached but declined screening may have been eligible to participate, which means that the enrolment rate is likely to be over-estimated in this sample. Only a limited amount of data were collected on potential participants who agreed to be screened (using the AUSDRISK tool), reducing the number of variables that could be examined in relation to enrolment rates. However, the model was able to correctly predict enrolment outcomes in 69% of individuals, suggesting that the variables examined were important predictors. Some of the screening questions, particularly those relating to physical activity levels and fruit and vegetable consumption were crude single item measures, and hence caution is required in interpreting these results. Further research is required to confirm these findings across a larger number of studies, using both quantitative and qualitative methods. In particular, qualitative interviews with eligible participants who decline to take part in diabetes prevention programs may provide important insights to complement these quantitative findings. This study was also unable to examine the contribution of individual versus GP/practice factors in influencing enrolment rates using multi-level analysis. This type of analysis was not possible in this study due to limited availability of data at the GP and practice level. Future research in the general practice setting should aim to examine predictors of enrolment at the practice, GP and individual level.

## Conclusions

Our findings highlight that engaging both primary health care providers and high risk individuals to participate in diabetes prevention programs remains an ongoing challenge. At the individual level, our findings suggest that some high risk groups including those with a family history of diabetes, a history of high blood glucose and physically inactive individuals are more likely to enrol in lifestyle intervention programs and may be worth targeting initially due to their high risk status and likely receptivity. However, other high risk groups are likely to require targeted strategies, in particular smokers and those born in countries with high diabetes risk. Further research is required to better understand factors influencing the uptake of diabetes prevention programs in primary health care, in particular the influence of individual, GP and practice factors.

## Competing interests

Authors declare that they have no competing interest in the conduct of this study.

## Authors’ contributions

RL conceived the analysis approach, undertook the analysis and wrote the first draft of the manuscript. SC and PV designed and implemented the SDPP and were responsible for data collection, management and interpretation. DD assisted with data management and cleaning. KV provided statistical advice and assistance. CR contributed to design and implementation of SDPP and the writing of the paper. All authors contributed to data interpretation and have read and approved the final manuscript.

## Pre-publication history

The pre-publication history for this paper can be accessed here:

http://www.biomedcentral.com/1471-2458/12/822/prepub

## References

[B1] WHO Preventing Chronic Disease: a vital investment WHO a global report 2005

[B2] Whiting DR IDF Diabetes Atlas: Global estimates of the prevalence of diabetes for 2011 and 2030 Diabetes Res Clin Pract 2011 94 3 311 321 10.1016/j.diabres.2011.10.029 22079683

[B3] Pan XR Effects of diet and exercise in preventing NIDDM in people with impaired glucose tolerance: The Da Qing IGT and diabetes study Diabetes Care 1997 20 4 537 544 10.2337/diacare.20.4.537 9096977

[B4] Tuomilehto J Prevention of type 2 diabetes mellitus by changes in lifestyle among subjects with impaired glucose tolerance N Engl J Med 2001 344 18 1343 1350 10.1056/NEJM200105033441801 11333990

[B5] Knowler WC Reduction in the incidence of type 2 diabetes with lifestyle intervention or metformin N Engl J Med 2002 346 6 393 403 11832527 10.1056/NEJMoa012512 PMC1370926

[B6] Kosaka K Noda M Kuzuya T Prevention of type 2 diabetes by lifestyle intervention: A Japanese trial in IGT males Diabetes Res Clin Pract 2005 67 2 152 162 10.1016/j.diabres.2004.06.010 15649575

[B7] Ramachandran A The Indian Diabetes Prevention Programme shows that lifestyle modification and metformin prevent type 2 diabetes in Asian Indian subjects with impaired glucose tolerance (IDPP-1) Diabetologia 2006 49 2 289 297 10.1007/s00125-005-0097-z 16391903

[B8] Yamaoka K Tango T Efficacy of lifestyle education to prevent type 2 diabetes: a meta-analysis of randomized controlled trials Diabetes Care 2005 28 11 2780 2786 10.2337/diacare.28.11.2780 16249558

[B9] Gillies CL Pharmacological and lifestyle interventions to prevent or delay type 2 diabetes in people with impaired glucose tolerance: Systematic review and meta-analysis Br Med J 2007 334 7588 299 302 10.1136/bmj.39063.689375.55 17237299 PMC1796695

[B10] Absetz P Type 2 diabetes prevention in the “real world”: One-year results of the GOAL implementation trial Diabetes Care 2007 30 10 2465 2470 10.2337/dc07-0171 17586741

[B11] Greaves CJ Motivational interviewing for modifying diabetes risk: A randomised controlled trial Br J Gen Pract 2008 58 553 535 540 10.3399/bjgp08X319648 18682011 PMC2566518

[B12] Kulzer B Prevention of diabetes self-management program (PREDIAS): effects on weight, metabolic risk factors, and behavioral outcomes Diabetes Care 2009 32 7 1143 1146 10.2337/dc08-2141 19509014 PMC2699739

[B13] Mensink M Study on lifestyle intervention and impaired glucose tolerance Maastricht (SLIM): Preliminary results after one year Int J Obes 2003 27 3 377 384 10.1038/sj.ijo.0802249 12629566

[B14] Laatikainen T Prevention of type 2 diabetes by lifestyle intervention in an Australian primary health care setting: Greater Green Triangle (GGT) Diabetes Prevention Project BMC Public Health 2007 7 249 10.1186/1471-2458-7-249 17877832 PMC2039742

[B15] Penn L Prevention of type 2 diabetes in adults with impaired glucose tolerance: the European Diabetes Prevention RCT in Newcastle upon Tyne, UK BMC Public Health 2009 9 342 10.1186/1471-2458-9-342 19758428 PMC2760530

[B16] Makrilakis K Implementation and effectiveness of the first community lifestyle intervention programme to prevent Type 2 diabetes in Greece. the DE-PLAN study Diabet Med 2010 27 4 459 465 10.1111/j.1464-5491.2010.02918.x 20536519

[B17] Saaristo T Lifestyle intervention for prevention of type 2 diabetes in primary health care: One-year follow-up of the finnish national diabetes prevention program (FIN-D2D) Diabetes Care 2010 33 10 2146 2151 10.2337/dc10-0410 20664020 PMC2945150

[B18] Almeida-Pititto B Predictive factors of non-deterioration of glucose tolerance following a 2-year behavioral intervention Diabetol Metab Syndr 2010 2 1 52 20673337 10.1186/1758-5996-2-52 PMC2919448

[B19] Bourn DM Impaired glucose tolerance and NIDDM: Does a lifestyle intervention program have an effect? Diabetes Care 1994 17 11 1311 1319 10.2337/diacare.17.11.1311 7821173

[B20] Page RC Can life-styles of subjects with impaired glucose tolerance be changed? A feasibility study Diabet Med 1992 9 6 562 566 10.1111/j.1464-5491.1992.tb01839.x 1643806

[B21] Payne WR Effect of a low–resource-intensive lifestyle modification program incorporating gymnasium-based and home-based resistance training on type 2 diabetes risk in Australian adults Diabetes Care 2008 31 12 2244 2250 10.2337/dc08-0152 18796621 PMC2584172

[B22] Schäfer S Lifestyle intervention in individuals with normal versus impaired glucose tolerance Eur J Clin Invest 2007 37 7 535 543 10.1111/j.1365-2362.2007.01820.x 17576204

[B23] Vanderwood KK Implementing a state-based cardiovascular disease and diabetes prevention program Diabetes Care 2010 33 12 2543 2545 10.2337/dc10-0862 20805260 PMC2992185

[B24] Colagiuri S The Sydney Diabetes Prevention Program: A community-based translational study BMC Public Health 2010 10 328 10.1186/1471-2458-10-328 20534170 PMC2898827

[B25] Chen L AUSDRISK: An Australian Type 2 Diabetes Risk Assessment Tool based on demographic, lifestyle and simple anthropometric measures Medical Journal of Australia 2010 192 4 197 202 20170456 10.5694/j.1326-5377.2010.tb03507.x

[B26] ABS 2033.0.55.001 - Census of Population and Housing: Socio-Economic Indexes for Areas (SEIFA), Australia - Data only 2006 http://www.abs.gov.au/ausstats/abs@.nsf/mf/2033.0.55.001/2006 [cited 2008 March]

[B27] Narayan KMV Randomized clinical trial of lifestyle interventions in Pima Indians: A pilot study Diabet Med 1998 15 1 66 72 10.1002/(SICI)1096-9136(199801)15:1<66::AID-DIA515>3.0.CO;2-A 9472866

[B28] Wang Z Hoy WE Si D Incidence of type 2 diabetes in Aboriginal Australians: An 11-year prospective cohort study BMC Public Health 2010 10 487 10.1186/1471-2458-10-487 20712905 PMC2931471

[B29] Gary-Webb TL Neighborhood Socioeconomic Status, Depression, and Health Status in the Look AHEAD (Action for Health in Diabetes) Study BMC Public Health 2011 11 349 10.1186/1471-2458-11-349 22182286 PMC3111582

[B30] Willi C Active smoking and the risk of type 2 diabetes: A systematic review and meta-analysis J Am Med Assoc 2007 298 22 2654 2664 10.1001/jama.298.22.2654 18073361

[B31] Chinn DJ Factors associated with non-participation in a physical activity promotion trial Public Health 2006 120 4 309 319 10.1016/j.puhe.2005.11.003 16473376

[B32] Robroek SJW Determinants of participation in worksite health promotion programmes: A systematic review Int J Behav Nutr Phys Act 2009 6 26 10.1186/1479-5868-6-26 19457246 PMC2698926

[B33] Diener-West M Sociodemographic and clinical predictors of participation in two randomized trials: Findings from the Collaborative Ocular Melanoma Study COMS Report No. 7 Control Clin Trials 2001 22 5 526 537 11578786 10.1016/s0197-2456(01)00157-x

[B34] Klosky JL Predictors of non-participation in a randomized intervention trial to reduce environmental tobacco smoke (ETS) exposure in pediatric cancer patients Pediatr Blood Cancer 2009 52 5 644 649 10.1002/pbc.21946 19156856 PMC2733242

[B35] Harris M Laws R Amoroso C Moving towards a more integrated approach to chronic disease prevention in Australian general practice Aust J Prim Health 2008 14 3 112 119

[B36] Wong KC Brown AM Li SCH AUSDRISK: Application in general practice Aust Fam Physician 2011 40 7 524 526 21743862

[B37] Glasgow R Does the chronic care model serve also as a template for improving prevention Milbank Q 2001 79 4 579 612 10.1111/1468-0009.00222 11789118 PMC2751207

[B38] Amoroso C The 45 year old health check - Feasibility and impact on practices and patient behaviour Aust Fam Physician 2009 38 5 358 362 19458808

[B39] Balasubramanian B Practice level approaches for behavioral counseling and patient health behaviors Am J Prev Med 2008 35 5S S407 S413 18929988 10.1016/j.amepre.2008.08.004

[B40] Etz R Bridging primary care practices and communities to promote healthy behaviors Am J Prev Med 2008 35 5S S390 S391 18929986 10.1016/j.amepre.2008.08.008

[B41] Krist AH An Electronic Linkage System for Health Behavior Counseling. Effect on Delivery of the 5A’s Am J Prev Med 2008 35 5 S350 S358 10.1016/j.amepre.2008.08.010 18929981

